# Global Changes in Unproductive Splicing and NMD Efficiency in Tumors

**DOI:** 10.32607/actanaturae.27892

**Published:** 2026

**Authors:** L. G. Zavileyskiy, A. A. Mironov, D. D. Pervouchine

**Affiliations:** Center for Molecular and Cellular Biology, Moscow, 121205 Russia; Faculty of Bioengineering and Bioinformatics, M.V. Lomonosov Moscow State University, Moscow, 119991 Russia

**Keywords:** Nonsense-mediated decay, unproductive splicing, regulation, NMD efficiency

## Abstract

The nonsense-mediated mRNA decay (NMD) pathway is a mRNA quality control mechanism which not only degrades deleterious transcripts but also orchestrates a large number of post-transcriptional regulatory programs through unproductive splicing. We have developed a robust metric derived from splicing quantification in the RNA-seq data to measure NMD efficiency at a sample level. We demonstrate that NMD efficiency varies substantially both between and within tissues, with the magnitude of the variation comparable to that observed upon knockdown of the core NMD factor UPF1. By analyzing TCGA cancer cohorts, we further show that, in many tumors, unproductive splicing events undergo coordinated changes towards either collective suppression or collective activation of NMD isoforms, which is indicative of global deregulation of the activity of the NMD pathway. Consistently, we observed a striking divergence of NMD efficiency in cancers from the tissue-specific baseline level, suggesting that tumors partially erase the NMD signature of their tissue of origin. The application of the developed metric to RNA-binding protein knockdowns made it possible to identify several novel potential regulators of NMD efficiency. In sum, this study provides a solid framework for quantifying NMD efficiency, describes its biological and clinical relevance, and opens new avenues for dissecting mechanisms of post-transcriptional gene expression regulation by the NMD pathway.

## INTRODUCTION

The nonsense-mediated mRNA decay (NMD) pathway is a mRNA surveillance system that selectively eliminates transcripts containing premature termination codons [1]. Such transcripts may result not only from nonsense or frameshift mutations but also from alternative splicing [2]. On the one hand, elimination of mRNAs with premature termination codons prevents the production of deleterious proteins. On the other hand, NMD acts together with alternative splicing as a widespread post-transcriptional mechanism of gene expression regulation, referred to as unproductive splicing [2, 3]. The activity of the NMD system exerts a substantial impact on the transcriptome composition and has been linked to diverse physiological processes and pathological conditions, such as cellular differentiation, lymphocyte development, stress response, genetic disorders, and cancers [4, 5, 6, 7, 8, 9].

Transcript-level determinants of NMD efficiency have been widely studied [11, 23, 24], yet the information on global, condition-specific NMD efficiency often remains contradictory. Early works used reporter constructs and monitored a small number of endogenous NMD targets, revealing apparent inter-tissue variability, but these estimates could suffer from gene-specific regulatory biases [22, 25]. Approaches based on allele-specific expression of transcripts carrying nonsense mutations concluded that NMD efficiency is largely uniform across tissues [23, 24], but a later study of the expression levels of NMD-sensitive isoforms and matched productive isoforms again reported non-random inter-tissue differences [26].

Motivated by these conflicting observations, we sought to develop a robust and sensitive metric for the quantitative assessment of NMD efficiency using RNA sequencing (RNA-seq) data. Here, we introduce an integral NMD efficiency metric based on unproductive splicing events (USE), i.e. local alternative splicing events that generate isoforms containing premature termination codons and are sensitive to NMD inhibition. USEs are far more abundant than heterozygous nonsense mutations and provide substantially greater statistical power. Estimates using this metric have confirmed that inter-tissue differences may reach up to 80% of the effect size observed in UPF1 knockdown. The application to cancer data demonstrated a correlation between the direction of changes in NMD efficiency in tumors and the direction of changes in most individual events. The metric further showed that tumors frequently lose the NMD efficiency levels characteristic of their tissue of origin; in several cancer types, this alteration correlates with unfavorable prognosis. Juxtaposition of USE splicing changes with the corresponding gene-expression changes allowed one to distinguish event-specific splicing regulation from global NMD efficiency shifts. By applying the developed metric to RNA-binding protein (RBP) knockdown experiments, we confirmed known NMD factors and proposed new candidate regulators of the NMD pathway.

## EXPERIMENTAL

**RNA sequencing data**

Transcriptomes of healthy and tumor human tissues from The Cancer Genome Atlas (TCGA) were downloaded from the dbGaP portal as alignments to the GRCh38 human genome assembly. Thirteen tumor cohorts containing at least 15 paired tumor-normal samples were selected (Supplementary Table S1). Only matched tumor-normal samples were used in differential splicing analysis. The transcriptomes of human tissues from the Genotype Tissue Expression (GTEx) V7 project were downloaded in FASTQ format from the dbGaP portal. We selected 10,100 samples with a read length of 75 nt, containing at least 20 million reads per sample (Supplementary Table S2). Transcriptomes of K562 and HepG2 cells subjected to RBP knockdowns or knockouts were downloaded from the ENCODE consortium website [27] as alignments to the GRCh38 human genome assembly. Perturbations performed in two biological replicates in one or both cell lines were selected (Supplementary Table S3). Transcriptomes of HeLa cells subjected to knockdowns of spliceosomal components were obtained from the Array Express repository under accession number E-MTAB-11202 and converted to the FASTQ format [28]. Each knockdown was represented by one biological replicate. Transcriptomes of HeLa cells subjected to knockdowns of UPF1, SMG6, SMG7, and double knockdown of SMG6 and SMG7, as well as rescue experiments, were obtained from the SRA repository under the accession number GSE86148 [29]. Reads from the samples in FASTQ format were mapped to the human genome version GRCh38 using STAR aligner v2.7.8a [30], with GENCODE 43 [31] annotation used as a reference. Gene expression levels were assessed using the FeatureCounts utility [32]. Differential gene expression in TCGA was analyzed using limma-voom from the edgeR package [33]. In RBP and NMD system inactivation experiments, differential expression was analyzed using DESeq2 [34].

**USE Catalog**

A catalog of annotated unproductive splicing events (USEs) was generated using NMDj utility [35] based on the ENSEMBL genome annotation (version 108). To identify novel USEs, transcriptomes of each TCGA sample were assembled using the StringTie v2.2.1 software with the “conservative” option [36], and the aggregated annotation across all samples was fed to NMDj. Additionally, skipping events of all constitutive exons flanked by constitutive introns were added to the list of novel events. Novel USEs whose characteristic intron sets of NMD transcripts overlapped with the intron sets of annotated USEs were removed in order to obtain non-redundant catalogs [35].

**Quantitative assessment of splicing**

The number of split reads supporting intron splicing was computed from short read alignments using the IPSA package with the default settings [37]. The splicing rate of each USE was characterized by the Ψ metric calculated using the NMDj package [35], defined as the ratio of the number a of split reads supporting the NMD isoform to the total number a + b of split reads supporting both NMD and the coding isoforms (the value Ψ = 0 indicates the absence of the NMD isoform). Only USEs with a + b > 15 in at least half of the samples in each comparison group were considered. Differential splicing analysis in TCGA and NMD inactivation experiments was estimated using the random-effects model implemented in the statsmodels.stats.meta_analysis module. The arcsine transformation was applied to Ψ values to stabilize variance. The within-sample variance s was estimated using the formula s =  1 /  (4n +  2), where n = a + b, and the between-sample variance was estimated using the non-iterative method proposed by DerSimonian and Laird [38].

Estimates of the group means and total variances were used to conduct z-tests for the significance of intergroup differences. For RBP perturbation experiments with few or no biological replicates, a and b values were pooled across all the replicates, and the statistical significance of Ψ differences between groups was assessed using the proportion test. To compare splicing rates between the knockdown and control conditions, ΔΨ = ΨKD – ΨC was used, where ΨKD and ΨC are the Ψ values in the knockdown and control, respectively. The ΔΨ values were averaged across all the inactivation experiments of the same RBP; the P values were calculated using the Stouffer’s z-score method.

**Assessment of NMD efficiency**

The following metric α was used to select core USEs specifically responding to inactivation of the NMD system, but not to that of individual RBPs. This metric was calculated as the product of the ΔΨ value between the RBP inactivation and the control, normalized by the standard deviation across all RBPs, and the z-score of the corresponding P-value. It was required that the ratio of the α value for NMD inactivation experiments to the largest α value for all the RBPs from the panel of perturbation experiments be not less than L (see below). For each data set (TCGA, GTEx, and ENCODE), core USEs were selected from the set of events for which the proportion of samples with a + b > 15 was at least N (see below), and the proportion of samples with 0 < Ψ < 1 was at least K (see below). For each core event, the Ψ values were transformed to z-scores across all the samples. The NMD efficiency metric of a sample was defined as the negative mean of z-transformed Ψ values across all the core events.

The parameters N, K, and L, as well as core USEs, were optimized separately for each dataset due to biological (tumor, tissue, perturbation) and technical (sequencing depth) heterogeneity. For TCGA: N > 0.5, K > 0.1 in at least 12 out of 13 cohorts, L > 0.3, 120 events were selected. For GTEx: N > 0.9, K > 0.9 across all tissues, 111 events were selected. For ENCODE: N > 0.7, K > 0.7 across all experiments, L > 0.2, 127 events were selected. Experiments in which less than 70% of core USEs met these criteria were excluded from further analysis.

**Survival analysis**

Patient survival data were obtained from the UCSC Xena platform [39]. The association between NMD efficiency and the overall survival was assessed using the Cox proportional hazards model. Within each tumor cohort, patients were stratified into two equal groups, with high and low NMD efficiency. Sex and age were included as covariates; age was divided into three equal-sized groups within each cohort.

**NMD efficiency and regulated unproductive splicing**

The following odds ratios were calculated to assess the abundance of specifically regulated USEs. For a given threshold value of the absolute ΔΨ value in tumors, the ratio of the number of USEs demonstrating opposite directions of splicing and expression changes and the number of USEs with co-directional changes among events with |ΔΨ| above and below the threshold value were compared. Only the genes with |log2 FC| > 0.3 and P < 0.05 were considered. The same estimates were calculated separately for positive and negative ΔΨ values to assess the dependence on the direction of changes in NMD efficiency.

**Statistical analysis**

**Fig. 1:**
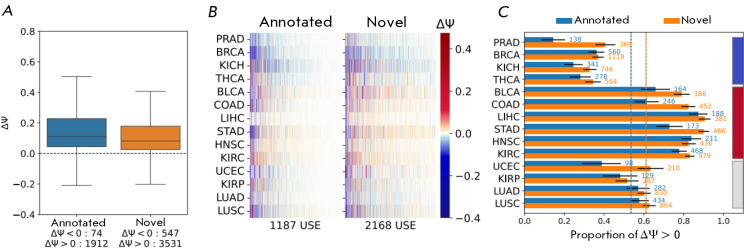
Changes in unproductive splicing upon NMD inactivation and in cancer cohorts from TCGA. (A) Changes in the splicing rates (ΔΨ) of USE in double knockdown of SMG6 and SMG7 in the HeLa cell line. (B) Changes in the splicing rates (ΔΨ) of USEs that are differentially spliced in at least one TCGA cohort. (C) The proportions of USEs with elevated NMD isoforms in tumor cohorts (ΔΨ > 0). Numbers to the right of the bars indicate the number of USEs. The dotted lines show the average proportions of USEs with elevated NMD isoforms across all cohorts. Error bars represent 95% confidence intervals. The blue, red, and gray colors represent tumor groups with the same direction of splicing alterations

All statistical tests were performed in Python 3.9.13. Bonferroni–Holm corrections for multiple testing were performed for differential expression and splicing analyses in TCGA and for RBP or NMD factor inactivation experiments separately for each tumor cohort or experiment, when assessing the significance of differences in NMD efficiency between tumor and normal tissue, when searching for associations between NMD efficiency and patient survival, and when assessing the enrichment of functional categories of RBPs using the GSEA method in a list of RBPs ranked by the magnitude of changes in NMD efficiency in the corresponding knockdown. Survival analysis was conducted using the lifelines module. Boxplot whiskers in all the figures correspond to the extreme points deviating from the median by no more than 1.5 interquartile range units. Error bars in Fig. 1 and Fig. 4, as well as Supplementary Fig. S3, correspond to 95% confidence intervals. In this paper, r and P denote the Pearson correlation coefficient and the adjusted P value, respectively; FC denotes the fold change in the gene expression levels. All nonparametric tests were performed using normal approximation with a continuity correction.

## RESULTS

**Unproductive splicing changes exhibit a preferred cancer-specific direction**

Our initial goal was to characterize global unproductive splicing changes in tumors. Using the NMDj tool [35], we assembled a catalog of unproductive splicing events (USE) that were either present in the ENSEMBL genome annotation (annotated) or inferred from the RNA-seq data for tumor transcriptomes (novel). We identified 1,986 annotated and 4,078 novel USEs responding significantly (P < 0.05) to NMD inhibition in the SMG6-SMG7 double knockdown experiments [29]. As expected, the relative abundance of the NMD isoform, as measured by the change in the percent-spliced-in (ΔΨ, NMD inhibition vs. control) metric, increased for the vast majority of events (more than 75%) (Fig. 1A).

USEs that had changed in the expected direction (i.e., increased the proportion of NMD isoform under NMD inactivation) were selected for further analysis. We found 1,187 annotated and 2,168 novel events that were differentially spliced in at least one tumor cohort (P < 0.05), with remarkably diverse patterns of both positive and negative changes (Fig. 1B). Next, we quantified the proportion of annotated and novel USEs with positive ΔΨ and tested statistically whether they differed from the respective average ΔΨ value across all cancer cohorts (Fig. 1C). Interestingly, most cancer types exhibited a preferred direction of unproductive splicing change: in PRAD, BRCA, KICH, and THCA, the proportion of NMD isoforms tended to decrease, whereas in BLCA, COAD, LIHC, STAD, HNSC, and KIRC it more often increased. This trend was observed for both the annotated and novel events; however, novel events showed a higher proportion of positive changes, which is consistent with their absence in genome annotations because of the low expression levels under normal conditions.

**USE-based metric robustly estimates the NMD efficiency**

The consistency in the direction of changes in unproductive splicing across tumors suggests that these alterations may be driven by global changes in the NMD pathway activity. To estimate NMD efficiency, we selected a core set of n = 120 USEs that (i) were expressed in at least 13 of the 14 analyzed TCGA cohorts, (ii) responded to NMD inhibition in the expected direction, i.e., by upregulation of NMD-sensitive transcripts, and (iii) were not sensitive to perturbations in the expression levels of RBP (in order to reduce the confounding effect of event-specific regulation, see Methods). For each sample, the NMD efficiency score was defined as the negative mean of z-transformed Ψ values of the selected core events.

**Fig. 2:**
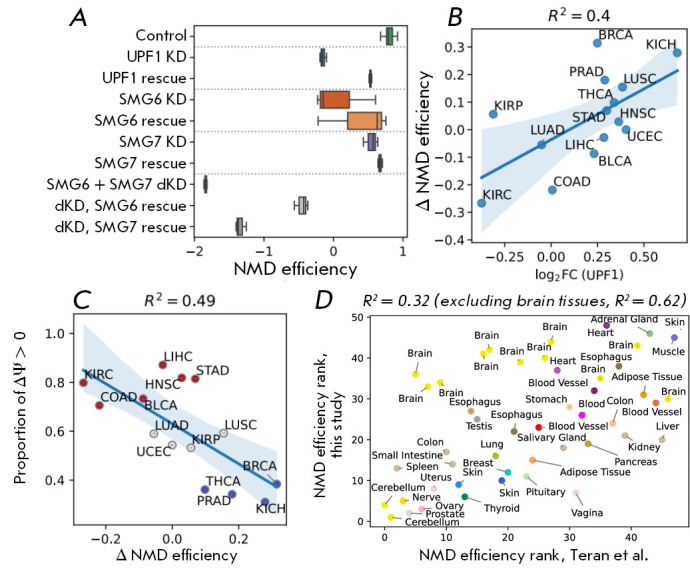
The estimation of NMD efficiency. (A) NMD efficiency upon knockdown and rescue of the expression of NMD factors in the HeLa cell line. (B) Correlation between changes in NMD efficiency and changes in UPF1 expression levels in tumors. (C) Correlation between changes in NMD efficiency and proportion of USEs with changes in positive splicing in tumors. Dot colors correspond to the colors of the tumor groups with the same direction of splicing changes in Fig. 1C. (D) Correlation between the NMD efficiency score in GTEx tissues from this study and the score based on nonsense mutations [23]

We validated this metric in several ways. First, the NMD efficiency scores were robust with respect to choosing a core set of events provided that it was large enough (n > 100) (Supplementary Fig. S1, see “Experimental”). Second, all the USEs in the core set showed highly coordinated splicing changes across TCGA samples (Supplementary Fig. S2). Third, the NMD efficiency score dropped in the experiments involving single and double knockdown of three key NMD factors (UPF1, SMG6, and SMG7), and recovered in the respective rescue experiments (Fig. 2A). Furthermore, changes in the NMD efficiency score in tumors vs. matched normal tissues positively correlated with changes in UPF1 expression levels (Fig. 2B). As expected, the proportion of USEs with collective positive ΔΨ changes in TCGA samples dropped from 80% to 20% with increasing NMD efficiency score (Fig. 2C). Finally, applying our metric to healthy tissues from the GTEx project revealed a strong correlation with an existing allele-specific estimate of NMD efficiency [23], in which the latter explained 62% of the variance in the former if brain samples were excluded (Fig. 2D). Notably, brain tissues deviated from the general linear trend in such a way that allele-specific estimates for different brain subregions were highly variable, while the NMD efficiency score introduced here was consistently high.

**NMD efficiency varies substantially both between and within human tissues**

While the existing NMD efficiency metrics allow one to perform relative comparisons across samples, they do not provide a sense of its absolute magnitude [23, 24, 26]. To address this issue, we introduced a reference scale by comparing the observed variation in NMD efficiency to the effect size for the knockdown of UPF1, the core factor of NMD, for which the expression level dropped by a factor of 3.5 [29]. With this scaling, the NMD efficiency score under unperturbed conditions was assumed to be 0, while it was assumed to be –1 under UPF1 depletion.

**Fig. 3:**
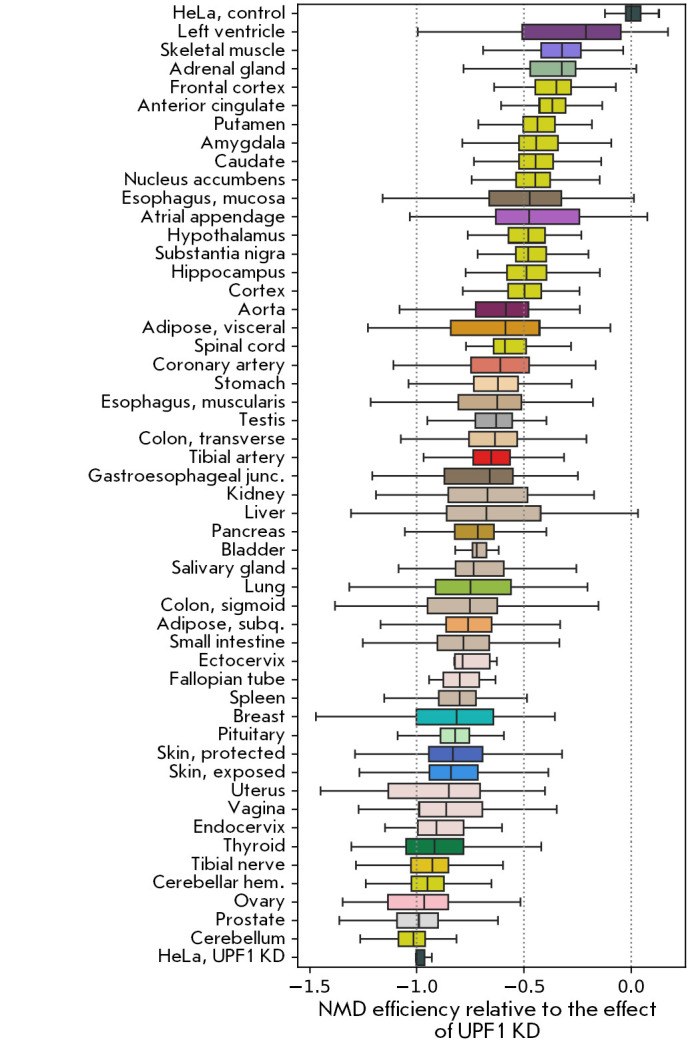
The distribution of NMD efficiencies in GTEx tissues relative to the magnitude of the effect of UPF1 knockdown. NMD efficiencies in the control and UPF1 knockdown HeLa cells were set to 0 and -1, respectively

As applied to the GTEx dataset, this transformed metric confirmed that NMD efficiency varies across tissues in a nonrandom manner, which is in line with recent findings [26] (Fig. 3). It is worth mentioning that 46% of the variance was attributed to the tissue factor (one-way ANOVA, P ≃ 0), and the difference between tissues with the highest (heart) and lowest (cerebellum) median NMD efficiency score was comparable to 80% of the effect of UPF1 knockdown. The inter-tissue variability was also remarkably high, with interquartile ranges reaching up to 40% of the effect of UPF1 knockdown in the heart, uterus, liver, and kidney. The least variable NMD efficiency levels were observed in brain subregions, but the difference between the median NMD efficiency levels in the brain cortex and the cerebellum was approximately half of the effect of the UPF1 knockdown, confirming that these two areas differ drastically in terms of NMD activity 26].

**NMD efficiency is associated with patient survival and regulated unproductive splicing**

**Fig. 4:**
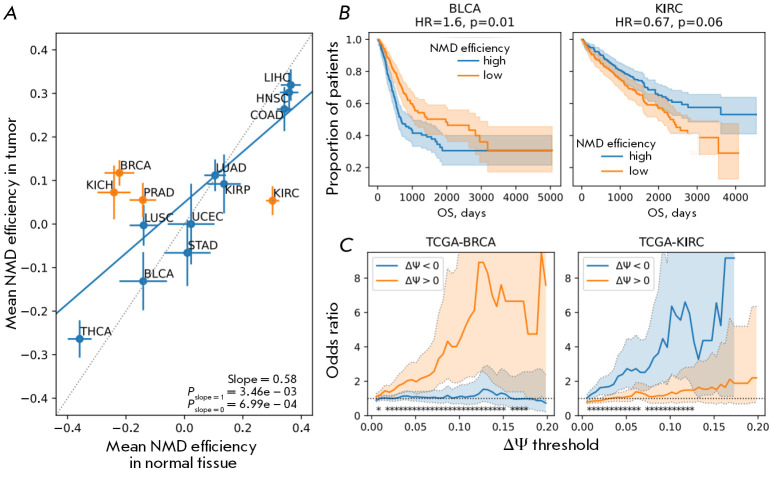
Association of NMD efficiency with patient survival and regulated unproductive splicing. (A) The least squares regression of the mean NMD efficiency in tumor versus that in normal tissue. Error bars represent standard errors of the mean. The dashed line corresponds to y = x. Tumors with a statistically significant difference in NMD efficiency are highlighted (P < 0.05, Wilcoxon test). (B) The Kaplan–Meier curves for patient groups with high and low NMD efficiency in tumors, for bladder carcinoma (BLCA) and clear cell renal cell carcinoma (KIRC). (C) Odds ratio of the oppositely directed changes in splicing and gene expression as a function of the threshold for the |ΔΨ| value and its sign. Asterisks denote a significant (P < 0.05) difference in odds ratios for positive and negative splicing changes

In order to examine the variability in NMD efficiency across tumors, we analyzed TCGA cohorts and found significant changes between NMD activity in cancer and the respective normal tissue in BRCA, PRAD, KICH, and KIRC (Fig. 4A). Remarkably, the direction of changes in NMD efficiency in cancer depended on the baseline efficiency of the corresponding normal tissue: it tends to increase in tissues with a low baseline NMD efficiency, while tending to decrease in tissues with a high baseline NMD efficiency. This led us to a nontrivial conclusion that tumors show a consistent trend toward erasing the tissue-specific NMD efficiency signature.

We next assessed the clinical relevance of alterations in NMD efficiency by relating it to patient survival. Significant associations with the overall survival rate were observed in BLCA and KIRC (Fig. 4B). Notably, the direction of these associations indicated the baseline NMD efficiency levels of normal tissue. In BLCA, which originally has low NMD efficiency, elevated NMD efficiency in tumors correlated with a poorer prognosis. Conversely, in KIRC, which exhibits high baseline NMD efficiency, reduction of its activity was associated with unfavorable outcomes (Fig. 4B). Together, these observations indicate that the greater the deviation of tumor NMD efficiency from the tissue-specific baseline, the worse the clinical prognosis; this trend persists for both positive and negative deviations.

As shown earlier, a substantial proportion of cancer-associated changes in unproductive splicing can be attributed to global shifts in NMD efficiency but some events may be regulated in a specific way. Event-specific regulation is expected to produce opposite changes in splicing and in the expression level of the host gene [40]. Using this negative association, we found that the proportion of specifically regulated USEs increased as we applied more stringent thresholds on the magnitude and statistical significance of splicing alterations (Supplementary Fig. S3). Moreover, these regulated USEs were dominant among the events that shift against the global direction imposed by the changes in NMD efficiency. For example, in BRCA, the cancer type with the strongest increase in NMD efficiency, the regulated USEs were significantly enriched among positive splicing changes, whereas an opposite pattern was observed in KIRC, which exhibited the strongest decline in NMD efficiency (Fig. 4C).

**The NMD efficiency metric recovers known and suggests novel regulators**

The core components of the NMD pathway including the UPF proteins (UPF1, UPF2, UPF3), the SMG kinases and adaptor proteins (SMG1, SMG5, SMG6, SMG7), and the exon junction complex (EIF4A3, RBM8A, MAGOH) have been characterized in considerable detail [41, 42, 43]. Yet, several recent studies suggest that the regulatory landscape of NMD extends beyond these canonical components [44, 45]. To explore this broader scope, we applied the NMD efficiency metric to a compendium of 587 RBP knockdown and knockout experiments and put forward a question as to which perturbations globally alter the NMD [28, 46].

**Fig. 5:**
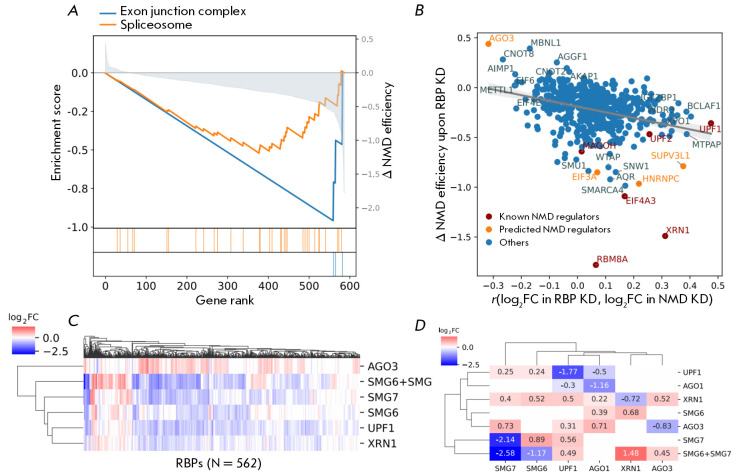
NMD efficiency in RBP inactivation experiments. (A) The gene set enrichment analysis (GSEA) plot for RBP ranked by the magnitude of changes in NMD efficiency in expression inactivation experiments. (B) Correlation between the changes in NMD efficiency and the similarity of transcriptional profiles upon RBP depletion and NMD inactivation. The latter was assessed using the Pearson correlation coefficient applied to the log2 fold change of the expression values (log2 FC) of differentially expressed genes in the RBP inactivation experiment and the NMD inactivation experiment (average over four NMD inactivation experiments). (C) Heat map of log2 FC for RBP genes upon knockdowns of the AGO3 and NMD factors. (D) A part of the heat map from (B) corresponding to changes in the expression of the AGO1, AGO3, and NMD factors. Statistically insignificant changes are not shown

Most perturbations reduced NMD efficiency, with exon junction complex components strongly being enriched among those with the largest effects (Fig. 5A). Decreased NMD efficiency was also frequently observed upon depletion of spliceosomal factors, which is consistent with the observations implicating spliceosomal components in the NMD control [45]. The pervasive tendency toward lower NMD efficiency upon depletion of almost any RBP is possibly indicative of the high interconnectivity of the RBP regulatory network, in which perturbing a single RBP can induce a cascade effect causing global deregulation of RNA processing and accumulation of NMD-sensitive transcripts that are normally suppressed by both NMD and splicing regulatory programs [47].

In order to be able to further characterize potential NMD regulators, we compared the gene expression changes that were induced by NMD inactivation with those observed after depletion of each RBP. Specifically, we related two quantities: (1) the changes in the NMD efficiency score under RBP depletion and (2) the similarity of transcriptional profiles upon RBP depletion and NMD inactivation (the average of four NMD inactivation experiments: UPF1, SMG6, SMG7 and SMG6 + SMG7). The latter was measured by the Pearson correlation coefficient of log2 fold change values of the expression of differentially expressed genes. RBPs whose inactivation reduced NMD efficiency tended to drive expression changes resembling those caused by NMD loss per se (Fig. 5B). SUPV3L1, HNRNPC, and EIF3A stood out among the RBPs not previously linked to NMD as the closest to bona fide NMD factors both by the NMD efficiency metric and by their transcriptomic signatures.

SUPV3L1 is a helicase best known for its role in mitochondrial RNA turnover, although small amounts of this protein also localize in the nucleus [48]. Our findings suggest that SUPV3L1 may also contribute to degradation of nuclear RNAs, since the core USEs events mostly take place in nuclear genes, which substantially respond to SUPV3L1 knockdown. HNRNPC binds poly-U tracts in mRNAs and is involved in alternative splicing, polyadenylation, and translation [49, 50, 51, 52]. HNRNPC is known to suppress the incorporation of numerous cryptic exons [53, 54]. Therefore, its impact on unproductive splicing may arise from event-specific regulation rather than direct involvement in NMD. EIF3A is the RNA-binding subunit of the eIF3 translation initiation complex, which was shown to interact physically with UPF1 and inhibit subsequent rounds of translation [55]. Knockdown of EIF3E, another eIF3 subunit, was previously shown to suppress NMD [56, 57]. Taken together, these observations point to reciprocal regulation between translation initiation and NMD.

We also identified putative negative regulators of NMD, including AGO3, whose depletion produced the strongest increase in NMD efficiency across all the tested RBPs. AGO3 is one of the four Argonaute genes involved in miRNA-guided translational repression [58]. Reporter assays mimicking RISC binding in 3’UTRs of NMD-sensitive transcripts previously showed that its paralog, AGO_2_, can inhibit NMD by suppressing the translation of NMD-sensitive transcripts, although it remains unclear to what extent it affects endogenous targets [59]. Our data indicate that AGO3 suppresses the degradation of a broad set of endogenous transcripts, leading to gene expression changes opposite to those caused by NMD inhibition (Fig. 5C). However, other components of the miRNA pathway (AGO1, DICER1, DROSHA, XPO5, and DGCR8) did not show similar effects, suggesting that the role played by AGO3 is not simply a consequence of canonical miRNA pathway activity. Notably, AGO3 knockdown---but not AGO1 knockdown---increased the expression of SMG7, which may partly account for the observed increase in NMD efficiency (Fig. 5D). These findings point to AGO3 as a candidate negative regulator of NMD and suggest that it may possess functions that are distinct from those of its paralogs.

## DISCUSSION AND CONCLUSIONS

Accurate assessment of the efficiency of the NMD pathway is essential for understanding its contribution to cancer biology. The expression levels of NMD factors are unsuitable as proxies for pathway activity, since core NMD components are subject to negative autoregulation [21]. Existing approaches for estimating NMD efficiency include reporter assays (e.g., globin reporters) [25], analysis of allele-specific expression in genes with heterozygous nonsense mutations [23, 24], and comparison of the expression levels of NMDsensitive and productive transcripts [26]. Each method has its limitations: reporter assays are low-throughput, allele-specific approaches rely on rare mutations that may be insufficiently represented in a sample [23, 26], and transcript-level quantification of NMD targets can be inaccurate for minor isoforms and sensitive to annotation errors [60].

In this study, we have developed an NMD efficiency metric based on the splicing rates in a core set of USEs. We used it to characterize NMD regulation across human tissues, cancers, and large-scale RBP perturbation datasets. Leveraging USEs as endogenous reporters of NMD provides a powerful alternative to existing transcript- or mutation-based metrics. Using this framework, we confirmed that NMD efficiency varies across human tissues in a nonrandom manner, quantified the magnitude of these differences relative to NMD inhibition experiments, revealed systematic shifts in NMD activity in tumors, and identified putative novel regulators of the NMD pathway.

An unexpected observation emerging from our analyses and previous reports is the substantial heterogeneity of NMD efficiency within individual tissues [26]. The main factor contributing to this variability is that tissues in GTEx and TCGA are mixtures of multiple cell types varying in their morphology, histology, and characteristic expression signatures [61]. Because NMD efficiency can vary between the individual cell types making up a tissue [62], the NMD efficiency metric is indicative not only of the tissue-specific NMD activity, but also its histological composition. Overall, these results emphasize the importance of considering tissue heterogeneity when interpreting any NMD efficiency estimates from RNA-seq.

In multiple cancer types, we observed a striking divergence of NMD efficiency from the tissue-specific baseline. Tissues with intrinsically low NMD efficiency (e.g., breast) tend to exhibit an increased NMD efficiency in tumors, whereas those with high baseline activity (e.g., kidney) often show reduced NMD efficiency. This “flattening” of the NMD profiles suggests that tumors partially erase the NMD signature of their tissue of origin. A possible explanation is that cancer cells acquire stem-like properties [67]. Stem and progenitor cells display NMD efficiencies distinct from those of mature, differentiated cells under many conditions [4, 68, 69], and tumor dedifferentiation may shift NMD toward these stem-associated states. Alternatively, tumor evolution imposes selective pressures such as chronic stress, altered translation demands, or immune system evasion, which may favor specific levels of NMD independent of the original tissue program. Therefore, deviations from the tissue-specific NMD level were associated with patient survival, suggesting that maintenance of physiological NMD activity may be unfavorable for tumor progression.

Together with the observed pattern of co-directional changes in unproductive splicing, our findings attest to the substantial deregulation of the NMD pathway in tumors, which undoubtedly contributes to cancer pathology.

## Abbreviations

NMD: nonsense-mediated decay
USE: unproductive splicing event
RBP: RNA-binding protein
RNA-seq: RNA sequencing
